# Effects of Screen Time and Season on Cardiovascular System Indicators in Primary Schoolchildren

**DOI:** 10.1134/S0362119721060086

**Published:** 2021-12-16

**Authors:** N. B. Pankova, I. B. Alchinova, O. I. Kovaleva, M. A. Lebedeva, N. N. Khlebnikova, A. B. Cherepov, L. A. Noskin, M. Yu. Karganov

**Affiliations:** 1 Institute of General Pathology and Pathophysiology, Moscow, Russia; 2 Konstantinov St. Petersburg Institute of Nuclear Physics, St. Petersburg, Russia

**Keywords:** blood pressure, heart rate variability, seasonal variability, children, primary school, computer screen time, hygienic standards

## Abstract

Indicators of the cardiovascular system, including heart rate (HR) and blood pressure (BP) variability parameters, were analyzed in primary school students with different computer screen times. The study included 4084 students of grades 1–4 (age 7–12 years) from 66 Moscow schools. The screen time at school and out of school was assessed by teachers, based on the national Sanitary Rules and Regulations: 0, no screen time; 1, screen time matching hygienic standards; 2, screen time at least twice greater than recommended. Physiological examinations were carried out by spiroarteriocardiorhythmography with a face mask, the conditions corresponding to the functional stress test (mild hypercapnia/hypoxia). Testing took place in spring and autumn (independent samples). Statistical data processing was performed using nonparametric criteria. It was revealed that the introduction of computer technologies in school lessons within the limits of hygienic standards was accompanied by an increase, within the normal range, of systolic BP in girls at the end of grade 2 and 4 and in boys at the beginning and end of grade 4. Screen time at least twice higher than the hygienic standard did not have an additional effect on BP, but provoked shifts in the function of autonomic regulation. Boys were more sensitive to the influence of this environmental factor. Their pattern of seasonal variability in total power (TP) of the HR variability spectrum was reversed compared to that of children who did not use computers at school; i.e., higher TP values were observed in spring. In grade 4, the process was accompanied by an increase in spontaneous arterial baroreflex sensitivity and a decrease in the relative power of the LF range in the variability spectrum of systolic BP. The changes were assumed to reflect the adaptive response to changes in educational environment.

## INTRODUCTION

Assessment of the regulatory systems, including both nervous (autonomic nervous) and humoral (neuroendocrine) systems, is of immense importance for evaluating the functional state of the human body during its growth and development [1]. An informative noninvasive method for the assessment is provided by continuous registration of heart rate (HR) parameters for a few minutes and a subsequent analysis of their variability with algorithms of spectral, geometric, and statistical analyses [2]. HR variability (HRV) is highly efficient in studying the effects of educational environmental factors on the growing body in children. The factors primarily include lack of exercise [3–5] and digital educational technologies, which have come into broadly use in the past years [6, 7].

It is clear that lack of exercise in schoolchildren only increases as computer and information technologies are introduced in the educational environment and reach the current stage of total digital transformation [8]. This was especially clearly seen in the period of online learning due to COVID-19, when the screen time increased almost thrice in schoolchildren [9, 10]. While a direct negative effect on the cardiovascular system has not been described for using computers and various gadgets [11], a greater portion of sedentary pastimes in a child’s behavioral structure increases the risk of cardiometabolic syndrome [12, 13]. In addition, lack of exercise is known to negatively affect the efficiency of the autonomic regulation of the cardiovascular system in children and adolescents [3–5] and to decrease their circadian variability [14].

For many years, we have performed monitoring studies of cardiovascular system parameters in children and adolescents, including analyses of HR and blood pressure (BP) variability indicators by spiroarteriocardiorhythmography (SACR) [15]. Significant changes in parameters were found to occur in Moscow first-year primary schoolchildren from 2002–2003 to 2014 [16]: the power of the LF range increased in the HRV spectrum with a respective increase in LF/HF. The sensitivity of the spontaneous arterial baroreflex remained unchanged as established by direct (from measurements in the breathing cycle) and indirect (from the α index) assessments [16]. In addition, seasonal variations were observed for systolic BP (sBP) and the LF/HF ratio in the HRV spectrum [17]. The changes occurring over half a year from spring to autumn and from autumn to spring were studied in primary and secondary school students and adults from 2004 to 2007. We observed that sBP decreased over an academic year and that LF/HF increased. However, opposite processes were detected in primary school students from 2016 to 2019; i.e., sBP increased over an academic year (from autumn to spring) and LF/HF decreased (from the second to fifth schooling year) [17].

Thus, substantial changes in cardiovascular system indicators (including their variability parameters, which characterize the state of regulatory systems) were observed in primary school students in the past years. We assumed that the changes are associated with computerization of education. The objective of this work was to check the assumption by analyzing the cardiovascular system indicators (including HRV and BP parameters) in primary school students differing in screen time. The parameters were monitored from 2006 to 2011, when computerization of education just started and new technologies were used in only some schools. Examinations by SACR were carried out in spring and autumn (two independent samples).

## METHODS

We used the data collected at Moscow schools as part of the “Schoolchildren’s Health” of the Moscow Health Department (from 2006 to 2011). In total, the data were from 4084 students of grades 1–4 (7–12 years of age) of 66 various schools; the students lacked verified pathology of the cardiovascular system and had no HR disorders detected in the study.

A SACR hardware and software complex (INTOKS, Russia) simultaneously and continuously records the ECG in standard lead I (with a subsequent HRV analysis), finger blood pressure by photoplethysmography (with a subsequent analysis of sBP and diastolic BP (dBP) variability), and respiratory parameters by using an ultrasonic air flow detector (the subject wears a spirometry mask). Maximal, minimal, and mean values are recorded along with the spectral parameters of HRV and BP (total spectral power (TP) and absolute and relative powers of the standard frequency ranges (HF, LF, and VLF)). It is possible additionally to calculate the indexes based on the spectral variability parameters (LF/HF of the HRV spectrum, centralization index = (VLF + LF)/HF of the HRV spectrum, and α index = (LF(HR)/LF(sBP))^1/2^) and the statistical and geometric variability parameters. Additional options include evaluation of the spontaneous arterial baroreflex sensitivity (using direct measurements in the breathing cycle) and cardiac performance indicators.

All examinations were performed in sitting subjects in the first half of the day. The recording duration was 2 min, thus precluding correct assessment and analysis of the VLF ranges in the HR and BP variability spectra.

The subjects were tested while wearing a spirometry mask and breathing spontaneously. The testing conditions are not indifferent to the subjects [15], and direct measurements of inhaled and exhaled air compositions have confirmed that wearing the mask simulates mild combined hypoxia/hypercapnia [18]. Examination while wearing a spirometry mask has been used as a functional stress test [19].

The screen time at school was estimated by teaches, based on the hygienic norms that were applicable in the study period (Sanitary Rules and Regulations 2.2.2/2.4.1340-03, http://www.consultant.ru/document/cons_doc_LAW_42836/). Screen times were ranked as follows: 0, no screen time; 1, screen time matching the sanitary requirements (15 min per day, during a single lesson); and 2, screen time at least twice greater than recommended. Out-of-school screen time was assessed by the teaches by questioning the parents, using the same ranking method.

Examinations were carried out two times per year (in October and from March to April). Examination time points were designated using two digits, of which the first shows the schooling year (1 to 4) and the second shows the season (1, autumn; 2, spring). All samples were independent. The sample sizes and general characteristics are summarized in Table 1.

**Table 1.** Sizes of the children samples examined at different time points

Testing time point	*n*	Rank 0		Rank 1		Rank 2	
		absolute	%	absolute	%	absolute	%
Girls							
1-1	513	94	18.3	403	78.6	16	3.1
1-2	310	55	17.7	242	78.1	13	4.2
2-1	208	78	37.5	122	58.7	8	3.8
2-2	129	68	52.7	52	40.3	9	7.0
3-1	332	121	36.4	204	61.4	7	2.1
3-2	369	174	47.2	180	48.8	15	4.1
4-1	392	126	32.1	260	66.3	6	1.5
4-2	350	135	38.6	205	58.6	10	2.9
Total	2603	851		1668		84	
Boys							
1-1	510	99	19.4	395	77.5	16	3.1
1-2	321	69	21.5	239	74.5	13	4.0
2-1	96	21	21.9	67	69.8	8	8.3
2-2	103	48	46.6	49	47.6	6	5.8
3-1	166	15	9.0	141	84.9	10	6.0
3-2	129	47	36.4	71	55.0	11	8.5
4-1	109	14	12.8	90	82.6	5	4.6
4-2	47	20	42.6	22	46.8	5	10.6
Total	1481	333		1074		74	

Time points are designated with two digits, the first indicating the schooling year (1 to 4) and the second, the season (1, autumn; 2, spring).

Data distributions were tested for normality by the Shapiro–Wilk test, which allows samples of up to 3000 participants (the Statistica 7.0 package). Based on the testing results, nonparametric tests were used in further statistical analyses. Between-group differences were evaluated by the Kruskal–Wallis *H*-test (multiple comparisons) or Mann–Whitney *U*-test (pairwise comparisons). Associations between indicators were assessed using Spearman’s correlation coefficient. Results are presented as the median and interquartile range (Me [Q1; Q3]) in tables and figures.

## RESULTS AND DISCUSSION

The data were generally similar to reference ranges used in medicine (heart rate, BP, and cardiac performance parameters) and reports from other studies (HRV, with due regard to the testing conditions with a spirometry mask on) for gender- and age-matched samples [20]. Significant between-group differences were not observed for respiratory parameters (tidal volume and breathing rate), cardiac performance parameters (stroke volume and cardiac output), spontaneous arterial baroreflex sensitivity (as measured directly in a breathing cycle), and statistical and geometric HRV parameters. As further analyses showed, the most informative parameters in terms of achieving our objective were sBP and the indicators that reflect activity of the autonomic regulation of BP (relative power of the LF range in the sBP spectrum) and HR (TP and the α index, which characterizes the spontaneous arterial baroreflex sensitivity) [21]. Only these indicators showed statistically significant between-group differences. Additional attention was paid to LF/HF in the HRV spectrum, because the LF/HF ratio is interpreted as an indicator of autonomic balance [2, 20] and shows seasonal variations [17]. Average values of the parameters are summarized in Table 2.

**Table 2.** Cardiovascular system indicators (median and interquartile range) in children at different testing time points

Testing time point	Girls	Boys
sBP, mm Hg		
1-1	101.0 [93.5; 106.6]	100.2 [92.3; 107.0]
1-2	96.9 [90.6; 103.6]^#^	97.8 [91.0; 104.1]^#^
2-1	102.1 [95.5; 110.8]^#^	106.4 [97.0; 116.9]^#,^ *
2-2	102.5 [86.7; 120.0]^#^	100.3 [89.3; 111.5]^#^
3-1	103.6 [96.5; 112.5]^#^	104.8 [97.0; 116.9]^#^
3-2	108.6 [97.3; 117.4]^#^	100.2 [90.7; 109.7]^#,^ *
4-1	106.6 [97.0; 116.1]	108.8 [100.1; 117.4]^#^
4-2	108.9 [99.7; 117.6]^#^	109.8 [92.3; 115.7]
Relative power (%) of the LF range in the power spectrum of sBP variation		
1-1	22.9 [17.3; 29.4]	23.0 [17.4; 31.3]
1-2	24.4 [18.1; 31.2]	26.5 [19.1; 32.9]^#,^ *
2-1	21.4 [13.4; 28.7]^#^	22.8 [18.0; 33.3]
2-2	21.5 [15.3; 30.3]	22.9 [15.3; 34.6]*
3-1	19.6 [13.2; 28.7]^#^	23.6 [15.4; 30.0]*
3-2	22.1 [16.0; 30.0]^#^	24.1 [17.4; 32.2]
4-1	22.8 [15.6; 30.6]	23.2 [17.0; 32.1]
4-2	22.2 [15.9; 31.5]	23.6 [17.1; 31.6]
α index, ms/mm Hg		
1-1	8.46 [5.80; 12.58]	8.59 [5.87; 12.27]
1-2	8.24 [6.30; 12.42]	8.70 [6.21; 12.74]
2-1	10.10 [7.06; 15.76]^#^	9.17 [6.61; 13.97]
2-2	11.37 [7.62; 15.20]	11.57 [6.78; 16.87]^#^
3-1	10.19 [6.89; 14.97]	11.27 [7.32; 15.59]
3-2	10.95 [7.45; 15.74]	10.04 [6.35; 14.34]
4-1	11.06 [7.53; 16.29]	11.02 [7.86; 15.42]^#^
4-2	9.61 [6.60; 14.40]	12.81 [7.40; 16.01]
Total power of the HRV spectrum, ms^2^		
1-1	3774 [2168; 6650]	3382 [1781; 6384]
1-2	4399 [2181; 7275]^#^	3708 [1860; 7246]
2-1	3855 [2223; 7524]	3050 [1503; 6933]
2-2	4080 [2203; 6209]	3807 [2017; 7639]
3-1	3367 [1890; 6257]^#^	3065 [1582; 7418]
3-2	3800 [2163; 7750]^#^	3137 [1636; 7034]*
4-1	3816 [2035; 7486]	4008 [2299; 6677]
4-2	3924 [2157; 6996]	4396 [2064; 7645]
LF/HF ratio of the HRV spectrum		
1-1	0.59 [0.33; 1.04]	0.67 [0.36; 1.14]*
1-2	0.59 [0.35; 1.03]	0.67 [0.39; 1.28]*
2-1	0.60 [0.32; 1.09]	0.68 [0.37; 1.22]
2-2	0.49 [0.26; 0.84]^#^	0.64 [0.33; 1.09]*
3-1	0.64 [0.35; 1.06]^#^	0.72 [0.32; 1.51]
3-2	0.61 [0.35; 1.06]	0.60 [0.31; 1.02]
4-1	0.63 [0.36; 1.15]	0.58 [0.29; 1.03]
4-2	0.58 [0.33; 1.02]	0.73 [0.34; 1.40]

Differences from (*) girls or (^#^) the previous time point were significant at *p* < 0.05 by the Mann–Whitney test.

Although independent samples were analyzed in this study, seasonal variation was observed for sBP, which decreased over an academic year from testing time point 1-1 to point 2-1 in girls and from point 1-1 to point 4-1 in boys. The changes coincide with the time-related changes that we observed in various age groups from 2004 to 2007. Seasonal variations in other indicators were not confirmed statistically, which is natural because individual variations of the indicators (not only those of the cardiovascular system) are usually rather high and mask the seasonal changes in analyses of average values from independent samples. Repeated measurements in the same sample and evaluation of the differences (Δ) in the indicators are therefore optimal to use in order to detect seasonal variations [17, 22].

Calculation of the coefficients of correlation showed that the indicators chosen for a detailed analysis correlate with the screen time at school, but not with out-of-school screen time (Table 3). As is seen from Table 3, a direct correlation between sBP and screen time at school was observed in girls at the ends of the first (point 1-2), second (point 2-2), and fourth (point 4-2) schooling years and in the beginning of the third year (point 3-1) and in boys in the fourth schooling year (points 4-1 and 4-2).

**Table 3.** Nonparametric Spearman’s coefficients of correlation between the indicators of the cardiovascular system and the screen time at school

Testing time point	sBP	LF% (sBP)	α index	TP	LF/HF
Girls					
1-1	0.054	0.044	0.060	0.072	0.054
1-2	**0.122**	–0.058	0.085	0.004	0.035
2-1	–0.069	**0.139**	–0.075	–0.039	–0.010
2-2	**0.262**	0.012	**–0.193**	–0.016	–0.022
3-1	**0.130**	0.007	–0.048	–0.076	–0.029
3-2	0.057	–0.002	0.077	–0.022	0.066
4-1	0.039	0.082	–0.090	–0.093	–0.065
4-2	**0.152**	**0.105**	**0.125**	0.081	–0.041
Boys					
1-1	**–0.121**	–0.032	0.023	0.056	0.003
1-2	0.004	0.059	0.032	–0.054	0.085
2-1	0.019	–0.097	–0.126	–0.063	0.056
2-2	0.085	0.178	–0.038	0.044	–0.035
3-1	0.035	0.101	–0.093	–0.144	0.079
3-2	0.089	0.117	–0.050	–0.061	0.157
4-1	**0.238**	0.055	**–0.254**	**–0.222**	0.057
4-2	**0.331**	**–0.469**	**0.419**	**0.319**	**–0.451**

Statistically significant coefficients (*p* < 0.05) are in bold.

**Fig. 1.**
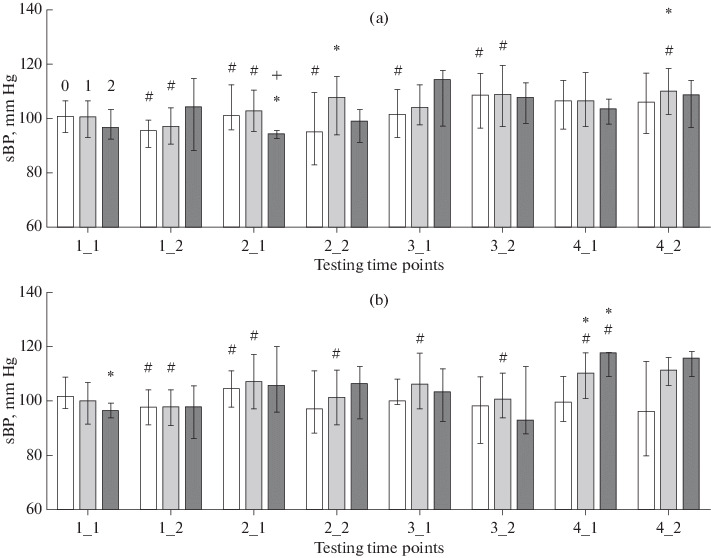
Systolic BP (sBP, mm Hg; median and interquartile range) in (a) girls and (b) boys. Testing time points are designated with two digits, the first indicating the schooling year and the second, the season (1, autumn; 2, spring). Children groups: 0, zero screen time; 1, screen time in accordance with sanitary standards; 2, screen time at least twice higher than the standard. Statistical significance of between-group differences: (*) difference from group 0 by the Kruskal–Wallis *H*-test (*p* < 0.05), (+) difference from group 1 by the Kruskal–Wallis H-test (*p* < 0.05), and (#) difference from the previous time point by the Mann–Whitney *U*-test (*p* < 0.05).

Higher sBP values in schoolchildren who used computers at school in compliance with sanitary regulations as compared with schoolchildren who did not use computers (differences between groups with screen time ranks 0 and 1) were observed in girls at testing time points 2-2 and 4-2 and in boys at time point 4-1 (Fig. 1). In girls, a group with a higher screen time (rank 2) had lower sBP values as compared with the groups with screen time ranks 0 and 1 in girls at the start of the second schooling year (point 2-1). In contrast, a boy group with rank 2 had higher sBP values at the start of the fourth schooling year (point 4-1).

A normal distribution was observed for a single parameter, sBP, in boys at time points 4-1 and 4-2. The parametric ANOVA algorithm was therefore additionally employed in sBP calculations in the boy groups at the time points. At time point 4-1, *F*(2, 106) = 3.2010, *p* = 0.039. Differences in mean sBP were evaluated by Tukey’s test: 0–1, 0.039 and 0–2, 0.235. The power of the ANOVA test was 1.000 for the three groups with the given mean sBP values and RMSSE calculated to be 14.17, even when assuming that the three groups are no more than *n* = 5 in size. Pairwise comparisons by Student’s test were as follows. Comparison 0–1: β = 1 – 0.773 = 0.227, *t* = 2.466 *df* = 102, *p* = 0.0153; comparison 0–2: β = 1 – 0.391 = 0.609, *t* = 1.995 *df* = 17, *p* = 0.0623. At time point 4-2, *F*(2, 44) = 4.4748, *p* = 0.017. Differences in mean sBP by Tukey’s test: 0–1, 0.020; 0–2, 0.144. The test power was 1.000 for the three groups with the given mean sBP values and RMSSE calculated to be 10.02, even when assuming that the three groups are no more than *n* = 5 in size. Pairwise comparisons by Student’s test were as follows. Comparison 0–1: β = 1 – 0.984 = 0.016, *t* = 2.689 *df* = 40, *p* = 0.0104; comparison 0–2: β = 1 – 0.819 = 0.181, *t* = 1.732 *df* = 23, *p* = 0.0966.

Thus, in boys examined at testing time points 4-1 and 4-2, group 1 with a screen time meeting the sanitary requirements had a higher mean sBP as compared with group 0 with zero screen time. Group 2 with an elevated screen time tended to differ from group 0 at both time points, but the difference was statistically nonsignificant. However, the high type II error probability makes it possible to assume that the problem is possible to overcome by increasing the size of group 2.

Russian sanitary requirements are more stringent than the requirements accepted in other countries [23], and the screen times that correspond to rank 2 are comparable with average screen times from similar studies [24]. It is still possible to compare our findings with the results reported by other research teams. There is still no convincing evidence that using computers per se negatively affects the basic parameters of the cardiovascular system [11] and their reactivity in loading tests [7], at least in students. However, additional information loading (associated with using a computer) has been shown to substantially activate the autonomic regulation of the cardiovascular system in 6- to 7-year-old children, shifting the balance towards sympathicotonia [25]. Similar shifts have been described in 9-year-old boys [26] and schoolchildren of the fifth grade [27]. A high screen time (total time at school and at home of more than 2 h) increases the risk of hypertension in healthy children and adolescents [28], and the condition is often accompanied by clinical signs of lipid metabolism disorders and a transition to obesity [29, 30]. An increase in screen time to extreme values in children with internet addiction correlates with autonomic dysfunction (sympathicotonia) of a central origin [31]. Thus, our finding that sBP tends to increase with the introduction of computer technologies into the education environment does not contradict the results reported by other research teams, although the indicator remains within the limits of normal for the gender and age when the sanitary standards are respected.

**Fig. 2.**
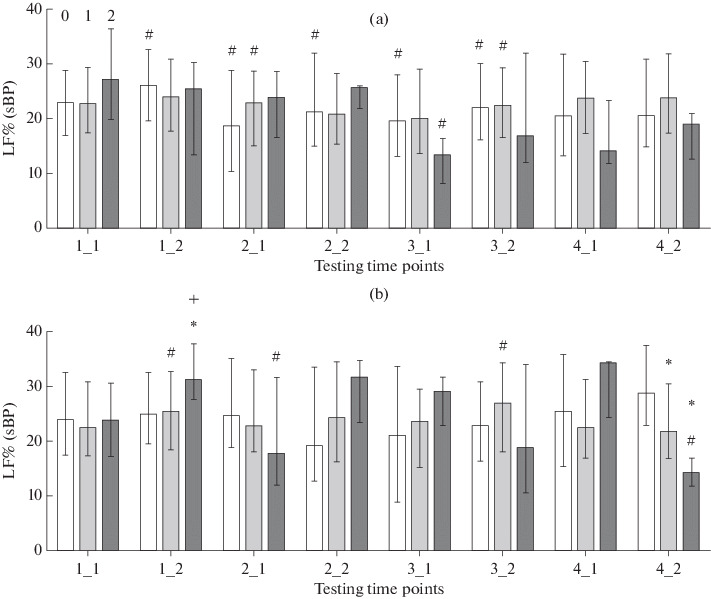
Relative power (%, median and interquartile range) of the LF range in the sBP variability spectrum in (a) girls and (b) boys. Designations are as in Fig. 1.

**Fig. 3.**
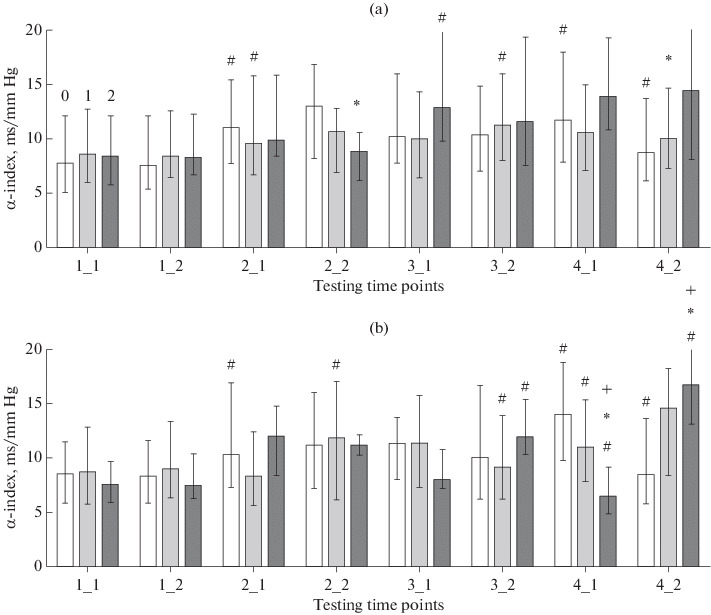
α index (ms/mm Hg, median and interquartile range) in (a) girls and (b) boys. Designations are as in Fig. 1.

Computerization of the educational environment is demanding to a child’s body [32]. The process acts essentially as a stress factor and induces an adaptive response in children [33, 34]. Traces of the response may change the developmental program of the child’s brain and cause morphological alterations in extreme cases [35]. An increase in screen time at an age of 6–7 years decreases the functional potential of the child’s body (as revealed using tests for physical development and its autonomic support) [24]. In our study, sBP remained within the limits of normal even in groups with higher screen times (rank 2). We therefore have no reason to think that use of computers in the educational environment is a risk factor to children’s health. However, significant shifts in several indicators were observed in the groups with rank 2, reflecting the developing adaptive response. The indicators include not only sBP, but also HR and sBP variability parameters as correlates of the functional state of autonomic regulatory systems. For example, a significant decrease in the relative power of the LF range in the sBP variability spectrum was observed in boys with higher screen times (rank 2) at the end of the fourth schooling year (time point 4-2) in addition to an increase in sBP (Fig. 2). The indicator is usually interpreted as a correlate of the functional activity level of brainstem centers [36] and baroreflex regulation [2, 20]. We used the α index, which reflects the spontaneous arterial baroreflex sensitivity and is calculated from the spectral parameters of HRV and BP variability [21]. In both boys and girls, higher α-index values were observed in the groups with higher screen times (rank 2) at time point 4-2 (Fig. 3). There are consequently reasons to assume that higher sBP values may be due to insufficient functional activity of the sympathetic regulation of vascular tone. We did not obtain similar results when analyzing the HRV spectral parameters, but the direction of the above changes coincides with that of changes in LF/HF, which were observed in the monitoring studies from 2016 to 2019 [17].

It should be noted that the α index showed seasonal variations in boys of the third and fourth schooling years and that opposite variations were observed in groups 0 (zero screen time) and 2 (high screen time) (Fig. 3).

Seasonal sBP variation, which manifested itself as a decrease in sBP during an academic year, was characteristic of the pooled sample (Table 2) and reproduced in the groups with rank 0 (zero screen time) within a range from testing time point 1-1 to point 3-1 in girls and from point 1-1 to point 2-1 in boys. In boys, the same variation pattern was additionally observed in the group with rank 1 (screen time within sanitary standards) in a range from point 1-1 to point 4-1 (Fig. 1). In girls, an opposite pattern was detected in the group with rank 1; i.e., higher sBP values were observed at the ends of the third and fourth schooling years (points 3-2 and 4-2). The groups with rank 2 (high screen time) did not display seasonal variation of sBP in both girls and boys.

**Fig. 4.**
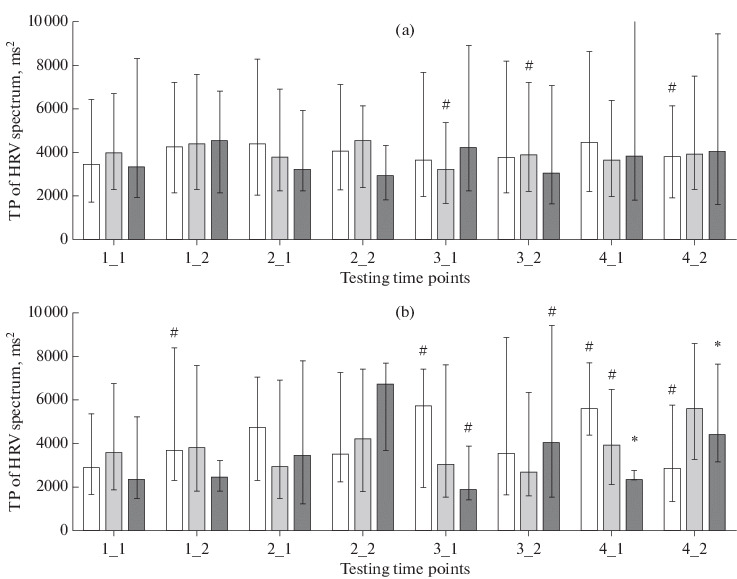
Total power (TP, ms^2^; median and interquartile range) of the HRV spectrum in (a) girls and (b) boys. Designations are as in Fig. 1.

However, a change in the pattern of seasonal variation in autonomic regulation indicators was most clearly seen in the analysis of the TP of the HRV spectrum. The TP is considered to be an integral parameter that reflects activity of the regulatory systems [2, 20]. In boys, opposite seasonal variations of TP were observed in the groups with ranks 0 (zero screen time) and 2 (high screen time), starting from the second schooling year. TP was higher in autumn in the former and spring in the latter (Fig. 4). In other words, the total level of autonomic activity decreases towards spring in boys learning without using computers, and, oppositely, increases in boys with higher screen times. The process was nonsignificant in girls in our study. Still opposite seasonal fluctuations of TP in children with different screen times indicate that computerization of the educational environment might be responsible for the change that was observed in the pattern of seasonal variation of cardiovascular indicators when comparing samples tested from 2004 to 2007 with samples tested from 2016 to 2019 [17].

Lack of exercise inevitably accompanies an increase in time that children spend looking at a computer screen or another gadget and certainly acts as an additional factor to induce shifts in the function of autonomic regulatory systems, including metabolic shifts towards anabolism [8–10]. The physical activity level was disregarded in our study. From 2006 to 2011, all Moscow schools followed the same standards with three gym classes per week according to recommendations. Specialized school that focusses on sports training were not included in the study.

It should be emphasized that conditions of a functional stress test [15] were used during testing that revealed the above changes in cardiovascular system indicators in primary school students with different screen times. Such conditions usually provoke a manifestation of latent (preclinical) shifts.

## CONCLUSIONS

Our screening study showed that computerization of education significantly affects the cardiovascular system indicators in primary school students examined in conditions of a functional stress test. In the case where computers were used in the educational environment (in classes at school) within the limits of sanitary standards, sBP increased in girls at the ends of the second and fourth schooling years and boys at the start and end of the fourth schooling year, still remaining within the limits of normal. Screen times at least twice higher than recommended by sanitary standards did not further affect BP, but provoked shifts in the function of autonomic regulatory systems. Boys were more sensitive to this environmental factor. An opposite pattern of seasonal variation in TP of the HRV spectrum was observed in boys with high screen times as compared with boys who did not use a computer at school, TP being higher in spring. In the fourth schooling year, the process was accompanied by an increase in spontaneous arterial baroreflex sensitivity and a decrease in the relative power of the LF range in the sBP variability spectrum. The above shifts reflect the adaptive response developing on exposure to changes in educational environment and are certainly beneficial. It is also important to note that the shifts do not go beyond the limits of normal (for the gender and age) even when examination is performed in conditions of a functional stress test.
